# Automatic Speaker Recognition System Based on Gaussian Mixture Models, Cepstral Analysis, and Genetic Selection of Distinctive Features

**DOI:** 10.3390/s22239370

**Published:** 2022-12-01

**Authors:** Kamil A. Kamiński, Andrzej P. Dobrowolski

**Affiliations:** 1 Institute of Optoelectronics, Military University of Technology, 2 Kaliski Street, 00-908 Warsaw, Poland; 2 BITRES Sp. z o.o., 9/2 Chałubiński Street, 02-004 Warsaw, Poland; 3 Faculty of Electronics, Military University of Technology, 2 Kaliski Street, 00-908 Warsaw, Poland

**Keywords:** cepstral analysis, Gaussian mixture model, genetic algorithms, speaker recognition, system identification, system verification, system comparison

## Abstract

This article presents the Automatic Speaker Recognition System (ASR System), which successfully resolves problems such as identification within an open set of speakers and the verification of speakers in difficult recording conditions similar to telephone transmission conditions. The article provides complete information on the architecture of the various internal processing modules of the ASR System. The speaker recognition system proposed in the article, has been compared very closely to other competing systems, achieving improved speaker identification and verification results, on known certified voice dataset. The ASR System owes this to the dual use of genetic algorithms both in the feature selection process and in the optimization of the system’s internal parameters. This was also influenced by the proprietary feature generation and corresponding classification process using Gaussian mixture models. This allowed the development of a system that makes an important contribution to the current state of the art in speaker recognition systems for telephone transmission applications with known speech coding standards.

## 1. Introduction

**Figure 1:**
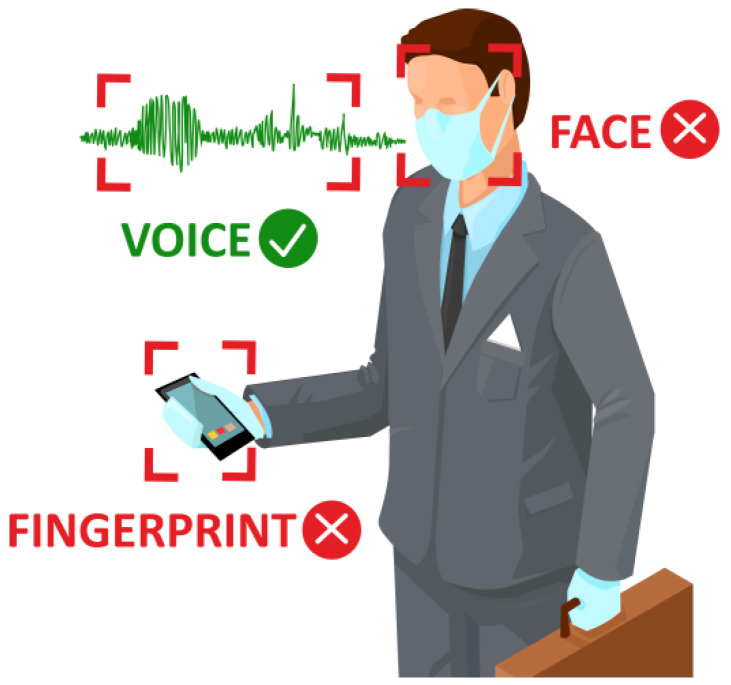
Advantage of voice biometrics over face and fingerprint biometrics during COVID-19 pandemic.

The human voice is an exceptional medium of communication. It cannot be lost, forgotten, or acquired without the conscious participation of its holder. Due to complicated processes, voice production strictly depends on the anatomy of the vocal organs, and the speech organ features significant ontogenetic differences. The voice as a biometric ID can be recorded by means of commonly used electronic devices, and its measurement is socially acceptable as it does not require direct contact. Nowadays, ‘touchless’ biometric IDs and solutions are extremely important and are still gaining importance during the time of crisis situations such as the COVID-19 pandemic. The current pandemic situation makes it impossible to verify identity by means of e.g., fingerprints, due to the common use of protective gloves. Face recognition is also hindered as people wear masks that cover a major part of the face. Thus, voice biometrics seems to be a good solution to that problem, as the protective elements mentioned above are irrelevant with respect to this method of biometric identification (Figure 1).

Although the intelligibility of the words uttered while wearing a mask may be slightly impaired, this probably does not have a big impact on the biometric features of the voice, which are mostly related to the anatomic structure of larynx. The larynx is the key element of the voice-producing part of the vocal organ. The larynx is composed of the laryngeal skeleton, ligaments, and muscles. There are internal and external muscles. The external ones are responsible for the larynx positioning; they move the larynx within all three planes and they participate actively in swallowing saliva or food. The larynx also moves during sound production. It rises during the production of high sounds, and it lowers during the production of low sounds. The voice production process would not be possible without the internal laryngeal muscles, especially the vocal muscles. The middle portion of the larynx includes vocal folds, vestibular folds, and the epiglottis [1].

When an individual is silent, the vocal muscles move the vocal folds apart so that the air flows freely through the glottis. When we whisper, our lungs produce so little pressure that the vocal folds do not vibrate, despite the fact that a whisper is an articulated sound as the noise enforced by a turbulent flow of air is modulated using the vocal tract. The situation is similar when voiceless sounds are articulated, as the vocal folds do not totally close the glottis, which also results in a noise-like turbulent flow of air. A different case is presented by the articulation of voiced sounds, accompanied by vocalization. This phenomenon occurs when the vocal folds come together and vibrate under pressure created by the air coming from the lungs and trying to force its way through the glottis. This process results in a laryngeal sound, and the fundamental frequency of the vocal fold vibrations is called the fundamental vocal frequency, which defines its tone. The frequency depends strictly on distinctive geometric features, i.e., the length and thickness of the vocal folds, glottis surface, and Rima glottidis surface, and on the mass of the vocal folds. Moreover, the fundamental frequency is determined by the vocal fold tension, which depends strictly on the speaker’s sex and age [1].

Due to some individual characters of laryngeal sound formation and its high dependence on the anatomical structure of the vocal organ, the laryngeal sound makes a perfect representation of an individual. A proper processing of the laryngeal sound enables the extraction of individual vocal features that ensure a high probability of individual identification, which opens up a range of possibilities for using the voice for biometric identification [1].

In the age of cell telephony, many banks, companies, and offices enable their users to perform various operations via telephone, by means of a so-called “security handshake”. An Automatic Speaker Recognition System fits very well in such a procedure. Thanks to the ASR System, one can avoid long-lasting verification questions that help with authenticating speakers. Moreover, if the ASR System is integrated with a speech recognition system, further increases in the security of remote operations are ensured via Text-Prompted Speaker Recognition. This helps to avoid impersonation if a voice recording of a given speaker is stolen. Thanks to vocal verification, one can avoid the need to hold additional security codes or to send personal details that can easily fall prey to unauthorized persons. The ASR System can also be used for the purposes of various access systems by enabling access to high security areas without the need to remember to carry a security ID card. To sum up, the ASR System may facilitate functioning in a contemporary IT society while increasing the level of the secure use of more and more devices that are equipped with biometric security systems. Future circulation of this technology may cause vocal biometrics to become a cheap and effective method of accessing security systems.

## 2. Related Work

This section presents an analysis of the latest scientific publications concerning speaker recognition. From an extensive collection of articles, only those were selected that described research for which the same voice dataset was used as the one used by the authors of this article. Such an approach makes it possible to verify whether the research work undertaken by the authors has resulted in tangible benefits relative to the competition.

The authors of the first publication under analysis [2] have conducted tests with the use of the NIST 2002 Speaker Recognition Evaluation (SRE) dataset [3] while making use of mel-frequency cepstrum coefficients (MFCC) and T-phase features in the distinctive feature vector. A Gaussian Mixture Model with Universal Background Model (GMM-UBM) method was used for classification. This enabled the achievement of an equal error rate of EER = 16.09% in the best variant of the proposed system.

The authors of subsequent papers [4,5,6,7] used the TIMIT database [8] for their research. The results of the studies conducted using the TIMIT database are the most widely available and thoroughly described, and so a comparison to other ASR systems using this database is described in detail in Section 4.5. In the present work, among others, the following were used for distinguishing features: MFCC [4,5,6,7] and power normalization cepstral coefficients (PNCC) [7]. In contrast, speaker classification in the present ASR System was based on GMM-UBM [4], latent factor analysis with Support Vector Machine (LFA-SVM) [5], Linear Discriminant Analysis (LDA) [6], Gaussian probabilistic LDA (GPLDA) [6], and extreme learning machine (ELM) [7].

The paper of [9], where research was performed based on the VCTK voice dataset [10] is described, is another publication that makes it possible to compare the results achieved. The authors used mel-frequency cepstral features based on Linear Predictive Coding (LPC) in the system architecture. Long Short-Term Memory Networks (LSTM) were used during classification, which resulted in EER = 5.10% in the best variant.

The analysis of the above-mentioned publications reveals a trend for using mel-frequency cepstral voice features that are also selectively used by the authors of this article. Moreover, GMMs with a huge number of Gaussian distributions are chiefly made use of by the competition for the purpose of classification. Attempts at applying deep neural networks (DNN) are also observed quite often during voice pattern classification [11]. The authors of this article make use of mel-frequency cepstral features and weighed cepstral features at the stage of generating distinctive features as well as GMM classifier, with a small number of Gaussian distributions for memory savings. A detailed description of particular stages can be found in Section 3. The test results achieved by the authors are provided in Section 4.2., where one can see an improvement in the results in comparison to the publications referred to above. For the NIST 2002 SRE dataset, EER was reduced by 14.57%; for the TIMIT dataset—by 0.14% (and by 10.19% for speech synthesized with the G.729 codec); and for the VCTK dataset—by 4.18% as compared to the competitive studies [2,6,9], respectively. In addition, for the TIMIT base, increases in identification rates (IRs) of 19% [4], 17.16% [5], and 0.87% (and 8.46% for speech noises using additive white Gaussian noise (AWGN)) were obtained [7], shown in Section 4.5. No results of the current research using the SNUV [12] dataset were found among the publications tackling the issue of speaker recognition. The remaining voice datasets described in Section 4 and that were used by the authors are proprietary voice datasets that were created due to the specific needs of experiments conducted by the authors. It should be noted that a cross-comparison of voice biometrics’ systems is not easy, due to the availability of multiple voice datasets, and only a comparison of systems on the same input data can be considered as being reliable. The following reviews of speaker recognition systems were also used when comparing the results to other ASR systems [13,14,15].

## 3. Methods

**Figure 2:**
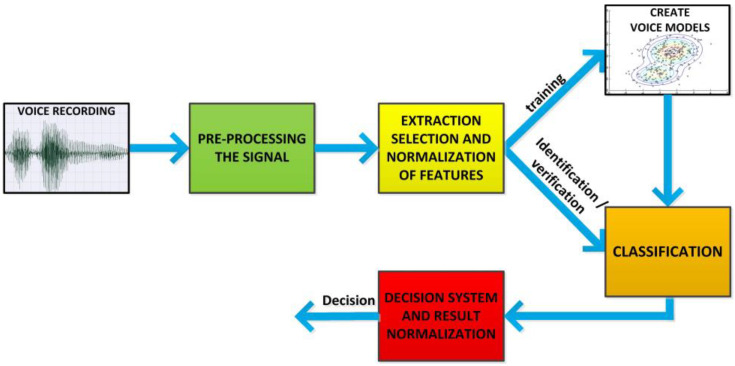
ASR System structure.

The structure of an Automatic Speaker Recognition System described in this article has been presented in Figure 2. Each of the modules presented are described in the further Sections of this article.

### 3.1. Speech Signal Acquisition

Voice signal acquisition is the first element of the System. This comprises signals that are subject to identification/verification, as well as signals that constitute training materials to make models of specific voices. When designing an ASR System, one should make a compromise between recording fidelity, which affects the preservation of individual features, and the amount of data processed, which affects computer memory resources and the rate of calculations. The ASR System subject to this article is dedicated for use under the conditions of limited quality that are prevalent in telephone transmission. A telephone tract has been made to transmit speech with as high fidelity as possible while sparing the band. According to numerous pieces of research, human speech is sufficiently comprehensible if the band reaches 3 kHz, which determines the assumed sampling rate within the telephone band. In order to avoid aliasing, the rate should, according to the sampling theorem, be at least twice higher than the maximum frequency of the incoming signal; this is why an excessive value of 8 kS/s was assumed [16].

The presented results of the ASR System testing were achieved in the most difficult possible operational conditions; this is why, if the voice dataset included signals recorded with a sampling frequency exceeding 8 kS/s, the signals were downsampled for the preservation of conditions that were similar to the telephone transmission conditions.

### 3.2. Signal Pre-Processing

The first element of signal pre-processing is its normalization, which involves two actions, namely, the removal of average value and scaling.

The removal of the average value from a digital speech signal is performed because of an imperfect acquisition process. In physical terms, a speech signal represents changes of acoustic pressure, this is why one can assume that its average value is zero. In a practical realization of digital speech processing, the value is almost always non-zero due to the processing of fragments of speech featuring a finite length.

Scaling is the second operation to which the signal is subject. It compensates signal non-matching to the scope of the converter while enabling the reinforcement of those speech fragments that were recorded too silently. In the case of a system dedicated to speaker identification/verification, regardless of the contents of utterance, it is not necessary to keep energy relations among the particular fragments of audio recording. This is why the authors perform scaling against a maximal signal value in order to avoid overdriving, and they use the available representation of numbers to be the maximum.

During audio recording analysis, it is important to eliminate silence, which is a natural element of almost every utterance. This operation enables a decrease in the number of signal frames to be analyzed, which contributes to how quickly a voice is recognized, and to the effectiveness of appropriate identification/verification, as only those frames are analyzed that are relevant from the point of view of speaker recognition. In the system presented, silence elimination takes place via the designation of the signal energy of the *i*-th *N* long frame, which is then normalized against a framework featuring the greatest energy *E_max_* from a given speaker’s utterance, according to the following equation:

(1) $$E_{i}=\frac{1}{E_{max}}\overset{n=N-1}{\underset{n=0}{\sum }}x^{2}\left(n\right)$$

Then, the achieved value of the normalized signal energy in frame *E_i_* is compared with the experimentally designated threshold *p_r_* (Equation (2)), which, in addition, depends on an average value of the energies of the particular frames *Ē*, which enables the appropriate elimination of silence in a diverse acoustic environment:

(2) $$E_{i}>p_{r}\bar{E}$$

Parameters related to frame length, shift, and threshold value were optimized with the use of a genetic algorithm [17].

Another element of signal pre-processing includes the limitation of sound components featuring the lowest frequencies (not heard by humans). For this purpose, a high-pass filter was used. Filter parameters were matched in the optimization process [17].

The subsequent stage of signal pre-processing includes signal segmentation in order to divide it into short (quasi-stationary) fragments called frames. This makes it possible to analyze each signal frame, from which separate vectors of distinctive features will be created at the further stage of the system operation. During the signal segmentation, the frame’s length as well as shift were subject to segmentation.

The segmentation process is related with windowing, i.e., signal multiplication by a time window *w*(*n*), the width of which determines the frame length *N*. It seems natural to apply a rectangular window in a time domain, as it does not distort the time signal. However, due to its redundancy, the time domain is not directly used for voice signal analysis. Frequency analysis is used for this purpose. The multiplication in the time domain operation corresponds to a convolution in frequency domain. The rectangular window spectrum is affected by a high level of sidelobes, which triggers the creation of strong artifacts in the signal analyzed, which in turn is related with the so-called spectrum leakage phenomenon. In order to minimize this adverse phenomenon, the Hamming window has been applied in the ASR System, which features a broader main lobe and lower levels of sidelobes:

(3) $$w\left(n\right)=0.54-0.46\;\cos\left(\frac{2\pi n}{N}\right)\begin{array}{ll} , & \end{array}0\leq n<N$$

In the speech recognition system, in which the semantic context of an utterance is the key element, it is justified to use windows of variable lengths, whereas in the case of speaker recognition systems which do not depend on speech content, there is no need for phonetic features-based segmentation, and this is why uniform segmentation is used.

The last step in the process of signal pre-processing involves the selection of its frames, which is important from the point of view of the ASR System. The above-discussed function of silence elimination is the first ‘rough’ stage of eliminating longer silent-speech fragments. The presented solution features three additional mechanisms of frame selection, which take place following the signal segmentation.

The first mechanism results from the desire to analyze laryngeal tone, which occurs only in voiced speech, which is why only ‘voiced frames’ are used during selection for the purpose of further analysis. In voiced fragments, the maxima in the frequency domain occur regularly (every period of the laryngeal tone), which cannot be said about voiceless speech fragments, which are similar to noise signals. The classification of voiced and voiceless speech fragments has been carried out with the help of an autocorrelation function [16]. The autocorrelation of signal frames is calculated according to the following equation:

(4) $$r\left(i\right)=\overset{N-1}{\underset{n=0}{\sum }}s\left(n\right)\;s\left(n+i\right),\begin{array}{ll} & i=0,\;1,\;2,\ldots \end{array}$$

where *s*(*n*) is a speech signal frame with the length of *N* samples. The occurrence of a periodical maxima in the autocorrelation function enables the defining of the resonance of the signal frame analyzed. The autocorrelation function achieves the highest value for a zero shift; however, this stripe is related to signal energy, which is why, when looking for resonant frames, one has to examine the second maximum *r_max_* of the autocorrelation function, which must be compared with the empirically calculated voicing threshold:

(5) $$r_{max}\geq p_{v}\;r\left(0\right)$$

The resonance threshold is another parameter that underwent optimization in the process of the development of the ASR System. The calculated autocorrelation function also serves to determine the speaker’s base frequency *F_0_*, which is a vital descriptor of the speech signal.

The re-detection of the speaker’s activity is another criterion applied when selecting representative signal frames, but this time, it involves the elimination of shorter silence fragments, the frame lengths and shifts of which are used for the further extraction of distinctive features. The process of choosing a threshold value of this criterion was also subject to optimization. Moreover, all of the hitherto mentioned system parameters were subject to multi-criteria optimization, with account being taken of their interrelations [17]. The signal frame is rejected if the following inequality is not fulfilled:

(6) $$P_{r}\geq p_{p}\;P_{s}$$

where *P_r_* is the power of the currently examined frame; *P_s_* is a statistical value of the average power of the frame; and *p_p_* is an empirically calculated threshold.

The last stage of frame selection takes place based on their noise level. This is possible thanks to a comparison of the value of the base frequency calculated by means of two independent methods—autocorrelation *F*_0*ac*_ and cepstral method *F*_0*c*_. Those two methods of calculating *F*_0_ have different resistances to signal noise. Making correct use of those characteristics enables us to define the signal frames that do not fulfil a defined quality criterion (Equation (7)) [18]. According to the literature [18], a calculation of the base frequency by means of the autocorrelation method is more exact than a calculation by means of the cepstral method, but the former one is less resistant to signal noise. Thus, the smaller the difference between base frequency determined by means of the two methods, the less noise in the signal frame concerned:

(7) $$\left|F_{0c}-F_{0ac}\right|\leq p_{f}\;\min\left(F_{0c},\;F_{0ac}\right)$$

Here, *p_f_* represents the optimized threshold value.

### 3.3. Generation of Distinctive Features

The generation of distinctive features, i.e., numerical descriptors representing the voices of particular speakers, is the key module of the biometric system. This stage is particularly important because any errors and shortcomings occurring at that point lower the capability for discriminating the parametrized voices of speakers, which cannot be made up for in the later stages of voice processing within the ASR System. The main goal of this stage of the operation is to transform the input waveform in order to achieve the smallest possible number of descriptors, including the most important information that characterize a given speaker’s voice, while minimizing their sensitivity to signal change, which is irrelevant from the point of view of ASR. In other words, minimizing the dependence on semantic contents or the parameters of the acoustic tract used during the acquisition of voice recordings.

Parametrization in the time domain is not effective in the case of speech signals, because despite its completeness, the signal—when considered in the time domain—features a very high redundancy. Further analysis in the frequency domain is far more effective from the point of view of this system. One of the reasons for such an approach is the inspiration for how the human hearing sense operates, which is developed in the course of evolution in order to correctly interpret the amplitude and frequency envelope of a speech signal by means of isolating the components featuring particular frequencies using the specialized structures of the internal ear [19].

**Figure 3:**
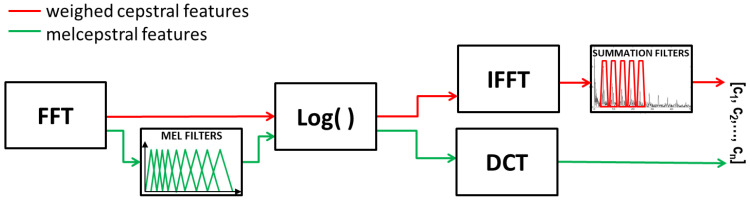
Diagram of generating distinctive features applied in the ASR System [17].

The frequency form of a speech signal is only a starting point for further parametrization. In the ASR System concerned, two types of descriptors were applied, which require further mathematical transformations of the amplitude spectrum, namely, weighed cepstral features and melcepstral features. The generation process of the features mentioned above are presented in Figure 3.

A detailed description of the particular mathematical transformations necessary to extract the considered distinctive features of the vocal signal are described below.

Following acquisition and initial processing, a speech signal assumes the form of a digital signal *s*(*n*), for *n* = 0, 1, …, *N* − 1, where *N* is the length of the signal frame considered. In order to make a signal transformation into the frequency domain, signal windowing takes place with the use of the window function *w*(*n*) (in the case of the ASR System concerned, it is the Hamming window) and Discrete Fourier Transform (DFT):

(8) $$S\left(k\right)=\overset{N-1}{\underset{n=0}{\sum }}s\left(n\right)w\left(n\right)W_{N}^{kn}$$

where $W_{N}=e^{-j2\pi /N}$ is the kernel of Fourier Transform. The input vector *s*(*n*) including *N* elements in the time domain is transformed into an output vector *S*(*k*), *k* = 0, 1, …, *N* − 1, which is a representation of the former in the frequency domain. In practical realization, one seeks that the number of samples be an iteration of 2, which allows for an implementation of the Fast Fourier Transform (FFT) algorithm.

A frequency analysis of the speech signal is more effective than a time domain analysis, and a direct analysis of the frequency spectrum enables the easy discrimination of semantic contents; however, extracting the ontogenetic character based on the spectrum envelope is still a difficult task. This is why, in further analysis, the signal will be subject to transformation to so-called cepstral time.

Cepstrum makes use of a logarithm operation, which in the case of complex numbers, is related to complications resulting from the need to ensure phase continuity. Since the phase analysis of sound is used by the hearing sense only to locate the source of the voice and does not carry any principal ontogenetic information, thus, in the case of speaker recognition, the so-called real cepstrum is used, which operates on the spectrum module and which for discrete signals, is reduced down to the following form:

(9) c(n)=IDFT(ln( | DFT(s(n) w(n)) | ))

and finally,

(10) $$c\left(n\right)=\frac{1}{N}\overset{N-1}{\underset{k=0}{\sum }}C\left(k\right)\;W_{N}^{-kn}=\frac{1}{N}\overset{N-1}{\underset{k=0}{\sum }}\ln\left(\left|\overset{N-1}{\underset{n=0}{\sum }}s\left(n\right)\;\;w\left(n\right)\;W_{N}^{kn}\right|\right)\;W_{N}^{-kn}\;$$

Due to the cyclicality of the Fourier transform kernel and the amplitude spectrum properties, the above logarithm from an amplitude spectrum module *C*(*k*) is cyclical and is described by the equation:

(11) C(k)=C(−k)=C(N−k)

This is an even function that ensures symmetry against the Y-axis, which is why its development features exclusively cosinusoidal (even) functions, and it does not matter whether, in the process of a reverse operation, a simple or inverse Fourier Transform is used. Due to this fact, a real cepstrum can clearly be interpreted as the spectrum of logarithmic amplitude spectrum [20]. The domain of such a transformation is called pseudo-time, and the X-axis is expressed in seconds.

When analyzing a speech signal spectrum, one can observe the occurrence of a fast-changing factor resulting from stimulation, and a slowly changing factor that modulates the amplitudes of particular impulses created as a result of the stimulation. Thanks to the application of a transformation in the form of an amplitude spectrum logarithm, it is possible to change the relationship of the two components from a multiplicative to an additive one. As a result of calculating the spectrum of such a signal (simple or inverse Fourier Transform), slowly the variable waveforms related to the vocal tract, which carry the semantic content, are located near to zero on the pseudo-time axis, while impulses related to the laryngeal sound occur near its period and repeat every period. The unequivocal determination of a starting point for the relevant information related with laryngeal sound is impossible, due to the fact that, in reality, the sound is not a single tone. This element was subject to optimization [17], and filtration in the domain of cepstral time is called liftering. 

**Figure 4:**
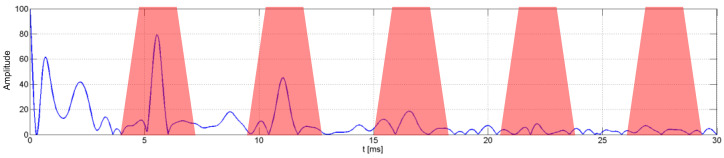
A method of setting weighted cepstral features from real cepstrum based on trapezoidal weighted function [17].

Cepstral analysis greatly facilitates the classification of speakers based on the obtained laryngeal sound peaks; however, in order to obtain the first type of distinctive features called weighed cepstral features, the authors of this article additionally applied summation filters in sub-bands [21]. The detection of maxima within the cepstrum in places foreseen by knowing a base frequency does not take place within this algorithm; what does take place is the summing up of all the points from this area with a certain weight. Sums around particular stripes, starting from the second maximum, are normalized to the sum of stripes surrounding the first maximum, which correspond to the base frequency. An idea for an algorithm applying a trapezoidal weighted function is presented in Figure 4. The type of filter applied, and the scope of summing, were subject to optimization [17]. 

Another parametrization method used by the authors, which is the most popular one in the case of speech signals, is the MFCC method [22]. This method involves the sub-band filtration of the speech signal with the support of band-pass filters distributed evenly on a mel-frequency scale.

The mel scale is inspired by natural mechanisms occurring in the human hearing organ, and reflects its nonlinear amplitude resolution. Although it is able to discriminate sounds within the range from 20 Hz to 16 kHz, the hearing organ is the most sensitive for the frequencies from 1 to 5 kHz. Thanks to the application of nonlinear frequency processing during speech signal analysis, it is possible to increase effective differentiation among particular frequencies. Low frequencies and logarithmic high frequencies are mapped (linear mapping) using the mel scale in huge approximation. Moreover, the scale allows for significant data reduction. Converting the amplitude spectrum to the mel scale is performed according to the following equation [23]:

(12) $$f_{mel}\left(f_{Hz}\right)=2595\;\log_{10}\left(1+\frac{f_{Hz}}{700}\right)$$

Frequency conversion takes place with the use of a bank of filters distributed in accordance with the mel scale described, using the above-mentioned equation. Particular filters possess triangular frequency characteristics. According to the literature, the filter shape has no significant importance if the filters overlap [24], which occurs in this case.

In order to conduct a linear mapping of the signal spectrum with the application of M filters, one has to carry out the following mapping:

(13) $$X_{i}=\overset{N-1}{\underset{k=1}{\sum }}\left(\left|S\left(k\right)\right|\;F_{i}\left(k\right)\right)\begin{array}{ll} , & i=1,\ldots ,M \end{array}$$

where *F_i_*(*k*) is the filter function in the *i*-th sub-band. This operation maps an *N*-point linear DFT spectrum to the *M*-point filtered spectrum in the mel scale. From 20 to 30 filters are used as a standard; however, numerous global studies on speaker recognition systems have proven that the use of a small number of filters has a negative impact on speaker recognition effectiveness.

Another stage during MFCC generation is to convert the spectrum amplitude to a logarithm scale, which facilitates the deconvolution of the voice-tract impulse response from laryngeal stimulation:

(14) $$X_{L_{i}}=\log X_{i}i=1,\ldots ,\;M$$

The last mapping of the features considered involves subjecting them to Discrete Cosine Transform (DCT), for the purposes of decorrelation, according to the following equation:

(15) $$MFCC_{j}=\overset{M}{\underset{i=1}{\sum }}X_{L_{i}}\;\cos\left[j\;\left(i-\frac{1}{2}\right)\frac{\pi }{M}\right]\begin{array}{ll} , & j=0,\ldots ,\;J \end{array}$$

where *M* is the number of mel filters; *J* is the number of MFCC coefficients; and *MFCC_j_* is the *j*-th melcepstral coefficient.

It is obvious that the initial melcepstral coefficients, that are closely related with the contents of the statement, would be rejected in further considerations, as in the case of resetting a portion of the signal located near zero on the cepstral time scale.

In an assessment of world researchers, in the case of speaker recognition, the best realization of MFCC parametrization is in the form proposed by Young [25], which gives a higher efficiency of speaker recognition than the original Davis and Memelstein method [22]; this is why it has been used for the purposes of our System. According to that idea, the definition of particular band ranges starts from calculating a range of frequencies ∆*f* according to the following equation:

(16) $$\Delta f=\frac{f_{g}-f_{d}}{M+1}$$

where *f_g_* is the upper frequency of the voice signal; *f_d_* is the lower frequency of the voice signal; and *M* is the number of filters.

That range is a fixed step that defines the filters’ middle frequencies, which are defined as follows:

(17) $$f_{sr,i}=f_{d}+i\cdot \Delta f,\begin{array}{ll} & i=1,\;2,\ldots ,\;M-1 \end{array}$$

The values of the above-mentioned frequencies are given in mels, which enable the even distribution of the filters on a perceptual scale, while preserving a nonlinear distribution within the frequency scale. The filter bands overlap so that each subsequent filter starts at the middle frequency of the previous filter.

### 3.4. Selection of Distinctive Features

At the feature generation stage, a maximally large set of distinctive features was extracted, which may be used for the purposes of the ASR System. According to global research, the use of a maximally large set of features does not always ensure that the best results are obtained [26,27,28]. Feature selection often enables the obtaining of greater or the same accuracy of classification for a reduced features’ vector, which in turn translates into a significant shortening of the calculation time and a simplifying of the classifier itself. Some features that are subject to evaluation may assume a form of measurement noise, which adversely affects the results of speaker recognition. There are features, however, that are strongly correlated with each other, which makes them dominate over the remaining part of the set and adversely affects the quality of classification.

The method of feature selection attempted by the authors uses a genetic algorithm (GA) [27]. This method enables an optimum set of features to be obtained, with account being taken of their synergy; however, this requires long-term calculations. In the initiation phase of the algorithm operation, the Shannon entropy of particular variables is calculated. In such a case, the variables are understood as being particular distinctive features and vectors of class membership. Entropy should be understood as a measure of disorder, which, for the case of one variable (18) constitutes a measure of its redundancy. If calculating the joint entropy (19), one takes account of the joint redundancy of both variables, and for totally independent variables, it assumes the value of the sum of entropies of particular variables:

(18) $$H\left(X\right)=-\underset{x\in X}{\sum }p\left(x\right)\log_{2}p\left(x\right)$$

(19) $$H\left(X,\;Y\right)=-\underset{x\in X}{\sum }\underset{y\in Y}{\sum }p\left(x,y\right)\log_{2}p\left(x,\;y\right)$$

The entropy values of particular variables and their joint entropies enable a level of mutual information to be defined according to the following equation [29]:

(20) I(X; Y)=H(X)+H(Y)−H(X, Y)

Mutual information allows for the use of a variable *X* (one of the distinctive features) for forecasting the value of a variable *Y* (another distinctive feature or one of the class membership vectors considered). In other words, it enables a definition of how much the knowledge of one variable may lower the uncertainty of another variable. The first two terms of Equation (20) attest to the “stability” of the features considered; the lower the redundancy of the features, the more certain the joint information and the higher its value. By subtracting the joint entropy of variables considered from the first two terms, one can easily obtain information about the level of the variables’ correlation. For totally dependent variables, the joint information equals 1, and for independent variables, it assumes the value of 0.

As a result, a vector of mutual information *I*(*yh_i_*; *y*) is created, which occurs among distinctive features *yh_i_* subject to selection and the class membership vector *y*, as well as a matrix of joint information among the features *I*(*yh_i_*; *yh_j_*). Due to the time-consuming nature of calculating the mutual information among the variables considered, in the case of the genetic algorithm presented, one calculates the initial data, which initiate the further operation of the algorithm. These data consist of mutual information calculated in a full set of features subject to selection. The initial data obtained are then used for each evaluation of the fitting of particular individuals (sets of individual features) within a population.

An initial population is created pseudo-randomly by generating chromosomes (vectors including the feature numbers drawn). Then, the chromosomes are assessed based on the fitting function (23), which includes the averaged mutual information between the features and the class membership vector (21), and between the mutually distinctive features (22) [29]:

(21) $$V=\frac{1}{N_{n}}\overset{N_{n}}{\underset{i=1}{\sum }}I\left(yh_{i};y\right)$$

(22) $$P=\frac{1}{N_{n}{}^{2}}\overset{N_{n}}{\underset{i=1}{\sum }}\overset{N_{n}}{\underset{j=1}{\sum }}I\left(yh_{i};yh_{j}\right)$$

(23) S=V−P

Not exceeding a set threshold value of a difference between a maximum value of the fitting function and its average value within a given situation is the criterion set by the authors for discontinuing further calculations. Calculations are also discontinued if a certain maximum number of generations has been achieved.

If the requirements for the discontinuation of calculations have not been satisfied, a further step of the algorithm involves the selection of the best fitted individuals. Prior to selection, the chromosomes are sorted according to the fitting functions, starting from the best fitted individuals. The selection is performed pseudo-randomly; however, it is with a preference for better fitted individuals according to the following equation [29]:

(24) $$g_{k}=round\left(\left(N-1\right)\frac{e^{ar_{k}}-1}{e^{a}-1}\right)+1$$

where *a* is a constant value; *r_k_* is a pseudo-random value from the range [0; 1], drawn for the *k*-th individual; *g_k_* is the index of the individual drawn, who was deemed well fitted and classified for undergoing further genetic operation (crossover).

**Figure 5:**
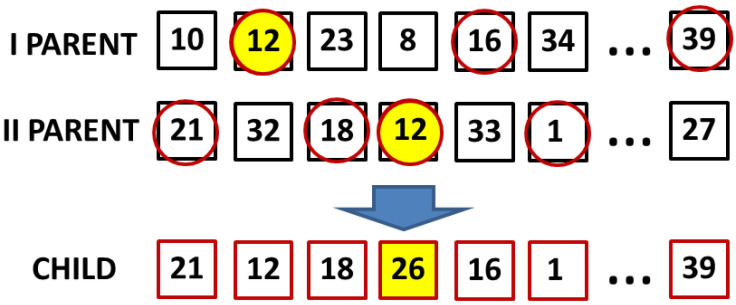
Depicting crossover and mutation operations within a genetic algorithm [17].

In crossover, for the purpose of establishing a set of features (a chromosome) of a new individual, one needs to crossbreed two parental chromosomes, so that in order to create *N* individuals of a new population, one needs to crossbreed 2*N* parental individuals. This is why for each *k*-th individual from the new population, two parental individuals are drawn, i.e., the selection function is realized twice (24). Crossover occurs in a multi-point way; namely, each feature of a new chromosome is matched pseudo-randomly from among the numbers of features occurring in a given index in a feature number vector of one of the two parental chromosomes (Figure 5). The drawing procedure is repeated until a new chromosome is obtained, the length of which is the same as that of the parental vectors. 

The above-mentioned mutation operation that takes place in the genetic algorithm is used in case features’ duplication during crossover. It is depicted in Figure 5 in the form of features marked with yellow circles. As a result of the genetic operations, a new population of individuals is created, which are subject to evaluation via the fitting function. The above-described operations are repeated until the termination condition is reached. As a result of feature selection by means of the genetic algorithm, we obtain a vector of features of the best fitted individual from the last generation.

Feature selection is related with an extra challenge, namely, an appropriate merger of the selected features, and a possibility for selecting prior to the merger and on the entire vector of descriptors. The authors have tested different variants of selection, as well as a merger of distinctive features, in order to choose a variant that enables the highest accuracy of speaker classification to be obtained [21].

### 3.5. Normalization of Distinctive Features

The normalization process is a further stage of distinctive feature processing. To this end, the authors have tested the following methods: cepstral mean subtraction (CMS); cepstral mean and variance normalization (CMVN); cepstral mean and variance normalization over a sliding window (WCMVN); and the so-called feature warping [30]. The last of the methods listed enabled the achievement of the highest effectiveness of correct identifications/verifications of speakers while minimizing the impact of additive noise and components related with the acoustic tone on the matrix of the feature vectors. This is why only that method will be described further.

The aim of warping is to map the observed distribution of distinctive features in a certain window of *N* observations, so that the final distribution of features is approximate to the assumed distribution *h*(*z*). Thanks to this approach, the resultant distributions of features may be more coherent in different environments of voice acquisition. Due to a multimodal character of speech, an ideal target distribution should also be multimodal and convergent with actual distribution of features of a given speaker; however, in this case, the authors have assumed a normal distribution:

(25) $$h\left(z\right)=\frac{1}{\sqrt{2\pi }}\exp\left(-\frac{z^{2}}{2}\right)$$

Such an approach may cause a non-optimal efficiency of the normalization process due to the simplification applied, which is an inspiration for further research in this respect.

It is worth pointing out that normalization of each feature takes place regardless of the other features, and that feature vectors from particular observations are already appropriately selected from among those that have not been created by a speech signal.

After selecting an appropriate target distribution, the normalization process may be started. In the respective window of *N* feature vectors, each of the features is sorted independently, in descending order. In order to define a single element of a warped feature while taking account of a feature located in the middle of a moving window, we calculate feature ranking in a sorted list. This is achieved in such a way that the most positive feature assumes the value of 1, and the most negative feature assumes the value of *N*. This ranking is then put into a lookup table. The lookup table can be used to map the rank of the sorted cepstral features into a warped feature using warping normal distribution. Considering the analysis window of *N* vectors and rank *R* of the middle distinctive feature in a current moving window, a lookup table, or warped feature elements can be defined by means of finding *m*:

(26) $$\frac{N+\frac{1}{2}-R}{N}=\int_{z=-\infty }^{m}h\left(z\right)dz$$

where *m* is the feature warped components. The warped value of *m* may be initially estimated by initially assuming the rank *R* = *N*, by calculating *m* by numerical integration and by repeating the process for each decreased *R* value.

### 3.6. Modeling the Speaker’s Voice

Thanks to the appropriate use of distinctive features that constitute a training dataset for the classifier, it is possible to create voice models that are economical in terms of memory and rich in terms of ontogenetic information at the same time. The GMMs used in the classification process constitute parametrical probability density-functions and are represented by the sums *C* of Gaussian distributions [31]. This enables the creation of vocal models that are relatively small in terms of memory but rich in ontogenetic information, by means of using a training dataset *X* = {*x*_1_, *x*_2_, …, *x_T_*}, where *T* means a number of *d*-sized vectors of distinctive features.

The initial values of the distribution parameters *λ* = {*x_i_*, *µ_i_*, Σ*_i_*} for *i* = 1, ..., *C* may be matched in a pseudo-random or determined way. Those parameters are: the expected values (*µ_i_*) and covariant matrices (Σ*_i_*), as well as the distribution weights (*w_i_*), while the sum of weights of all the distributions equals 1. Next, we calculate the probability density function of the occurrence of *d*-dimensional vectors of distinctive features originating from the training dataset of a given speaker in the created model of his/her voice. This function may be approximated by means of a weighted sum *C* of Gaussian distributions, and for a single observation t, it assumes the form of [32]:

(27) $$p\left(x_{t}|\lambda \right)=\overset{C}{\underset{i=1}{\sum }}w_{i}N\left(x_{t},\mu_{i},\Sigma_{i}\right)$$

The *N* function that describes a *d*-dimensional Gaussian distribution is defined by equation [32]:

(28) $$N\left(x_{t},\mu_{i},\Sigma_{i}\right)=\frac{\exp\left\{-\frac{1}{2}{\left(x_{t}-\mu_{i}\right)}^{\prime }\Sigma_{i}^{-1}\left(x_{t}-\mu_{i}\right)\right\}}{{\left(2\pi \right)}^{d/2}|\Sigma_{i}{|}^{1/2}}$$

During GMMs training, their parameters are estimated so that they match to training dataset *X*. This most often takes place via the estimation of maximum likelihood (ML), which is a product of the probability density function for the considered observation vectors [32].

(29) $$p\left(X|\lambda \right)=\overset{T}{\underset{t=1}{\prod }}p\left(x_{t}|\lambda \right)\;$$

The assumption behind the method is that *p*(*X|*$\bar{\lambda }$) ≥ *p*(*X|λ*), where model *λ* constitutes the initiation data for a new model $\bar{\lambda }$. Due to the numerous feature vectors used during model training, which for the same speaker are not equal to one another, the probability function (Equation (29)) is nonlinear and has a number of maxima, which frustrates its direct maximization. That is why the maximization of the probability function takes place through iteration, and an estimation of the model’s parameters takes place in accordance with the expectation maximization (EM) algorithm [33]. In practical realization, an auxiliary function is computed [32]:

(30) $$Q\left(\lambda ,\bar{\lambda }\right)=\overset{C}{\underset{i=1}{\sum }}\overset{T}{\underset{t=1}{\sum }}p\left(i|x_{t},\lambda \right)\log\left[{\bar{w}}_{i}N\left(x_{t},{\bar{\mu }}_{i},{\bar{\Sigma }}_{i}\right)\right]$$

where *p*(*i|x_t_*,*λ*) means the probability a posteriori of the occurrence of the *i*-th distribution in model *λ*, when feature vector *x_t_* is observed. According to the assumption of the EM algorithm, if inequality *Q*(*λ*,$\bar{\lambda }$) ≥ *Q*(*λ*,*λ*) occurs, then also *p*(*X|*$\bar{\lambda }$) ≥ *p*(*X|λ*).

The operation of the EM algorithm involves an iterative repetition of two steps. The first one is the estimation of the probability value *p*(*i|x_t_*,*λ*) (Equation (31)) [32], and the second one is maximization, which enables defining the parameters of the new model $\bar{\lambda }=\left\{{\bar{w}}_{i},{\bar{\mu }}_{i},{\bar{\Sigma }}_{i}\right\}\;$(Equations (32)–(34)) [32], which maximizes the function described by means of Equation (30). Each subsequent step makes use of quantities arrived at in the previous step. The process of model training is terminated in a lack of adequate increments of the probability function, or if a maximum number of iterations has been reached:

Estimation step

(31) $$p\left(i|x_{t},\lambda \right)=\frac{w_{i}N\left(x_{t},\mu_{i},\Sigma_{i}\right)}{\sum_{k=1}^{C}w_{k}N\left(x_{t},\mu_{k},\Sigma_{k}\right)}$$

Maximization step

(32) $${\bar{w}}_{i}=\frac{1}{T}\overset{T}{\underset{t=1}{\sum }}p\left(i|x_{t},\lambda \right)$$

(33) $${\bar{\mu }}_{i}=\frac{\sum_{t=1}^{T}p\left(i|x_{t},\lambda \right)x_{t}}{\sum_{t=1}^{T}p\left(i|x_{t},\lambda \right)}$$

(34) $${\bar{\Sigma }}_{i}=\frac{\sum_{t=1}^{T}p\left(i|x_{t},\lambda \right)\left(x_{t}-\mu_{i}\right){\left(x_{t}-\mu_{i}\right)}^{\prime }}{\sum_{t=1}^{T}p\left(i|x_{t},\lambda \right)}\;$$

**Figure 6:**
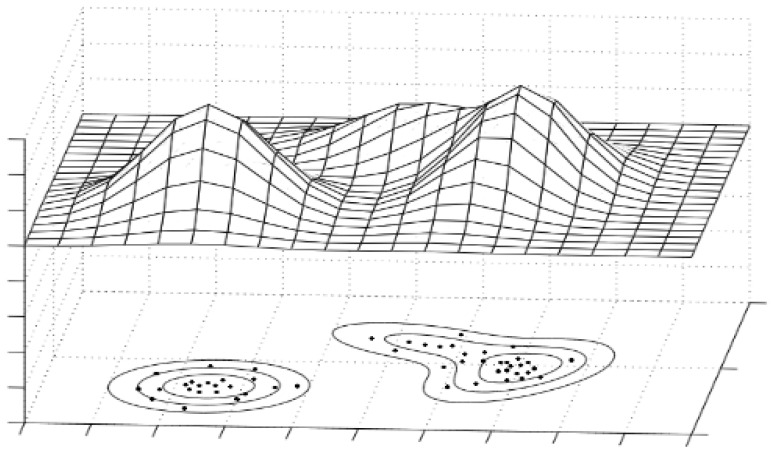
An example of modeling 2-dimensional training dataset by a weighed sum of 3 Gaussian distributions [17].

In order to depict a result achieved in the GMM training process, a distinctive feature vector has been limited to two dimensions, and models have been presented in a three-dimensional drawing (Figure 6). Each black point means a single multidimensional feature (in this case, a two-dimensional one) from each observation.

During speaker identification, a decision is made as to which speaker represented by the voice models *λ_k_* (for *k* = 1, …, *N*, where *N* is a number of voices in a given dataset), the recognized fragment of voice represented by the *X* set of distinctive features vector most probably belongs. To this end firstly, through a discrimination function *g_k_*(*X*) (Equation (37)), the conditional probability is calculated for each model, to attest that a specific model *λ_k_* represents the specific vectors of distinctive features *X* [32]:

(35) $$g_{k}\left(X\right)=p\left(\lambda_{k}|X\right)$$

Using Bayes’ theorem [32],

(36) $$p\left(\lambda_{k}|X\right)=\frac{p\left(\lambda_{k}\right)p\left(X|\lambda_{k}\right)}{p\left(X\right)}$$

where *p*(*X|λ_k_*) is a likelihood function originating from speaker model (29) and means a probability that a recognized *X* set of vectors is represented by a voice model *λ_k_*. Moreover, *p*(*λ_k_*) represents the statistical popularity of the voice in the dataset, and as each voice is equally probable, then *p*(*λ_k_*) = 1/*N. p*(*X*) is a probability of occurrence of a given feature vector X in a speech signal, and it is the same for each of the voice models. The probability is used for normalization purposes, and in the case of using the ranking only (according to criterion 40), when looking for the most probable model, it can be ignored. The discrimination function assumes the form of [32]:

(37) $$g_{k}\left(X\right)=p\left(X|\lambda_{k}\right)$$

The most probable voice model selection takes place on a ranking basis in accordance with criterion [32]:

(38) $$k^{*}=\arg\;\max g_{k}\left(X\right)$$

Finally, in practical realization, a log-likelihood value is determined, which enables a change of multiplicative relationship between subsequent observations to an additive one, and the criterion assumes the form of [32]:

(39) $$lk^{*}=\arg\underset{1\leq k\leq N}{\max}\overset{T}{\underset{t=1}{\sum }}\log\;p\left(x_{t}|\lambda_{k}\right)$$

where the probability *p*(*x_t_*|*λ_k_*) is determined according to Equation (27).

The process of classification with the use of GMMs is also related with a universal background model (UBM), which is created based on training data coming from various classes [34,35]. In the case of speaker recognition, we will call it a universal vocal model, and the training data used during the creation of its weighed mixture of Gaussian distributions includes the audio recordings of various speakers. The model has two main applications. The first one is to use it as initiating data when creating models of particular speakers. Thanks to such an approach, it is possible to train the model with a smaller number of iterations, as the model does not start from strongly divergent initial data. Moreover, as shown in the literature and in experiments conducted by the authors [35], thanks to the use of UBM in the ASR System, it is possible to achieve a better effectiveness of the correct identification/verification of speakers. The deterministic method of the matching initial values of a speaker’s voice model presented in this article is realized based on the GMM-UBM algorithm [36]. The authors used the above-mentioned algorithm and created a universal model of voices in numerous variants of limiting a voice cohort set included in the UBM training dataset. One of the approaches to limiting the cohort set involved the creation of a sex-independent and a sex-dependent UBM.

According to the experiments conducted by [36], if there is an additional attribute in the form of information about the probable sex of the recognized speaker, the chance of its correct identification/verification increases. This may be of vital importance for criminology, where UBM profiling is possible via training within a limited dataset of speakers or recordings originating from a given type of devices. 

A universal voice model may also be used in a decision-making system for the normalization of the achieved probability results, and in clearer terms—of the logarithm of probability (43) to make sure that the testing signal comes from a given speaker [36].

### 3.7. Decision-Making System

In the decision-making system within the automated speaker-recognition system, there are two hypotheses about probability where the distinctive features of an utterance analyzed actually occur in a given speaker’s statistical model. Those hypotheses may be formulated as follows:

- *H*_0_ (zero hypothesis)—voice signal *X* comes from speaker *k*,
- *H*_1_ (alternative hypothesis)—voice signal *X* comes from another speaker ~*k* from the population.

A decision regarding whether a voice signal *X* comes from speaker k or from another speaker ~*k* depends on the relationship of probabilities of the above-mentioned hypotheses and comparing it with a detection threshold *θ*. If we assume that the zero hypothesis is represented by model *λ_hyp_*, and that the alternative hypothesis is represented by model $\lambda_{\bar{hyp}}$, then the relation appears as follows:

(40) $$\Lambda \left(X\right)=\frac{p(X|\lambda_{hyp})}{p(X|\lambda_{\bar{hyp}})}>\theta$$

The above equation is called the Neyman–Pearson lemma or a likelihood ratio test (LRT) [37]. The likelihood quotient (40) is often given in a logarithmic (additive) form:

(41) $$\Lambda \left(X\right)=\log\left(\frac{p\left(X|\lambda_{hyp}\right)}{p\left(X|\lambda_{\bar{hyp}}\right)}\right)=\log p\left(X|\lambda_{hyp}\right)-\log p\left(X|\lambda_{\bar{hyp}}\right)>\theta$$

As a hypothesis, one can be assured of a result of Equation (39), where the GMM classifier created by the authors looks for a maximum value of the sum of probability density logarithms, which points to an existence of the feature vector *x_t_* in speaker model *λ_k_*. According to the above, this relationship may be presented as follows:

(42) $$\log p\left(X|\lambda_{hyp}\right)=\underset{1\leq k\leq N}{\max}\overset{T}{\underset{t=1}{\sum }}\log p\left(x_{t}|\lambda_{k}\right)$$

where *N* is the number of all voices in the dataset; and *T* is the number of distinctive feature vectors extracted from a recognizable speech signal. The ultimate form of the Neyman–Pearson lemma is as follows:

(43) $$\Lambda \left(X\right)=\log p(X|\lambda_{hyp})-\log p(X|\lambda_{\bar{hyp}})>\theta$$

The logarithm value of the likelihood of the alternative hypothesis $\log p(X|\lambda_{\bar{hyp}})$ in the presented System is calculated by means of a direct use of the likelihood logarithm obtained thanks to the created UBM.

(44) $$\log p(X|\lambda_{\bar{hyp}})=\log p(X|\lambda_{UBM})$$

### 3.8. Normalization of the Speaker Recognition Result

The normalization of the identification/verification result is the last stage of processing in the ASR System under consideration. To this end, the authors have tested the following normalization methods [38]:

- Test normalization—takes place online; test recording is verified against the declared speaker model and a group of other cohort models, followed by assigning an average and deviation from those results to a speaker under consideration;
- Zero normalization—the model is verified against initial utterances that do not come from the speaker modeled, followed by assigning an average of and deviation from those results to a speaker under consideration;
- Symmetric normalization—symmetric normalization calculates an average value of the normalized result of zero and test normalizations;
- Combined normalization—a combination of zero and test normalizations that assume that the results of the zero and test normalizations are independent random variations;
- Zero-time-normalization—is a combination of zero and time normalizations, during which time normalization takes place in the first place, followed by the zero normalization of the achieved verification results;
- Time-zero normalization—is a combination of zero and time normalizations, during which zero normalization takes place first and is followed by the time normalization of the achieved verification results.

The experiments conducted by the authors showed that the best way for the normalization of results in the case of the ASR System under consideration is combined normalization. In the course of that normalization, the results were subject to transformation according to the following equation [38]:

(45) $$C=\frac{T+Z}{2}\sim N\left(\frac{\mu_{Z}+\mu_{T}}{2}\frac{\delta_{Z}^{2}+\delta_{T}^{2}}{4}\right)$$

where *µ_Z_* and *µ_T_* are the subsequent average values resulting from the zero and time normalizations; and *δ_Z_* and *δ_T_* are standard deviations from those normalizations. Moreover, an appropriate matching of models making a cohort that takes place in the determination of the terms of Equation (45) is a vital element of the above-mentioned normalization.

## 4. Results and Discussion

The authors of this article have described experiments aiming to verify the correct operation of an automated speaker-recognition system. To this end, a series of tests was performed on a full dataset of voices available to the authors (1529 speakers). The datasets used during the experiments included four commercial datasets (NIST 2002 SRE [3], TIMIT [8], SNUV [12], VCTK [10]) and four proprietary datasets created for the purpose of optimization and verification of the System described here (intonation dataset [21], dataset with recordings of digits and numbers [31], multi-session vocal dataset [39], and dataset of recordings made with the use of, among others, a throat microphone in tanker headphones [40]). In order to carry out the above-described experiments, all the recordings from the voice datasets available have been subject to downsampling to a sampling rate of 8 kS/s, which is the same as the sampling rate used in telephony, while increasing the use of the System concerned. During the implementation of this ASR System, the authors’ and some built-in functions of the MATLAB environment were used. The time-consuming calculations during optimization were an unquestionable difficulty in the presented research. In order to accelerate them, the authors used the MATLAB environment toolkit, enabling the performance of parallel computing (Parallel Computing Toolbox). For optimization, among others, an appropriately adapted function of the Optimalization Toolbox of the MATLAB environment was used. The tests presented in the article were carried out on the final and optimized version of the presented ASR System.

In order to simulate all possible variants influencing the outcome of the decision system, authorized user authentication attempts were made during the experiments, which allowed for specification of the level of true acceptances—TAs, and false rejections—FRs. Attempts were also made to authenticate a potential intruder, which made it possible to measure the level of false acceptances—FAs, and true rejections—TRs. For an authorized user-authentication situation, the test speech signal was from the same *n*-th user in the dataset from which the *n*-th training signal was used to create the voice model to be compared came from. On the other hand, when a potential intruder was authenticated, the test signal came from the next speaker from the dataset (*n* + 1), and was compared with the voice model of the *n*-th speaker.

### 4.1. ASR System Effectiveness Test in Train and Test Segment Length Function

The first experiment was to test the impact of speech signal length on the effectiveness of speaker identification and verification. Tests were carried out for various lengths of both train and test segments. During the tests, GMMs were described by means of a single normal distribution for each speaker, without the use of a universal voice model, as data initiating the process of voice model generation.

**Figure 7:**
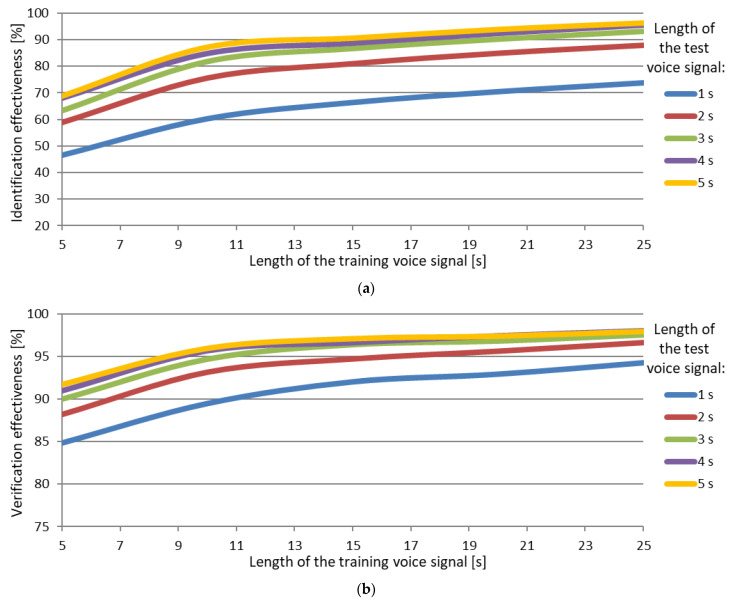
Speaker identification and verification effectiveness for variable lengths of speech signal, (**a**) Identification; (**b**) Verification.

Based on the analysis of the results obtained (Figure 7), one can conclude that the System is very effective in recognizing voice signals, the lengths of which are not shorter than 3 sec, and below this length, the System effectiveness drops clearly. This length is similar to the threshold time, which is the time necessary for a human being to correctly interpret a voice heard, especially if the voice comes unexpectedly.

### 4.2. Results of Speaker Equal-Error Rate in Particular Voice Datasets

Another experiment involved a series of tests to check the equal error rates achieved by the ASR System, in particular, the voice datasets (Table 1).

### 4.3. Effect of Coding Applied in Telephony on Speaker Recognition Effectiveness

The table below presents the impact of a typical coding situation applied in telephony on speaker recognition effectiveness. The following phonic coding standards have been used during the tests, including coded speech signals [34,41]: G.711; GSM 06.10; G 723.1; SPEEX. A broader description of the applied coding types has been presented in the authors’ publication [41]. During the tests, the authors made use of external libraries (SoX [42], Speex [43], and G.723.1 Speech Coder and Decoder library [44]). All the libraries were integrated into one application, which enabled the establishment of a converter for the purposes of the above-mentioned coding standards. Table 2 shows the identification accuracy—I, and the verification accuracy—V, while the results for the speech codec matching-conditions for the training and test signals are marked in bold.

When analyzing Table 2, one can conclude that an optimized version of the ASR System works fine, even with coding applied. One exception is where the coding system is not well-matched, which would be an extremely rare case in real, dedicated applications.

### 4.4. Speaker Identification in an Open Set of Voices

The last test to verify the quality of the ASR System is the depiction of the receiver operating characteristic (ROC) curve and the detection error tradeoff (DET) curve. Moreover, the authors calculated the value of the area underneath the curve (AUC), as well as the equal error rate (EER), which enables the determination of the quality of the decision-making system.

In order to conduct that test, the dataset of voices (1529) was divided in half, while keeping balance when dividing particular components of voice datasets, as well as keeping balance in terms of the division into genders. From the first half of the dataset, test signals and voice models were created, which allowed for the TA and FR results to be obtained. On the other hand, only test signals were created from the second half of the dataset, which made it possible to obtain the FA and TR results.

**Figure 8:**
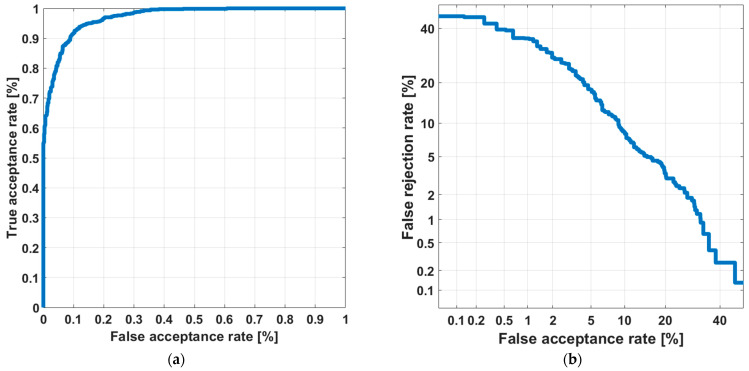
Results of speaker identification in an open set of voices, (**a**) ROC curve; (**b**) DET curve.

When analyzing the results (Figure 8), one can see that the obtained results are satisfying. The AUC value obtained equals 0.98 and the EER value equals 9.2%.

### 4.5. Validation of the ASR System Effectiveness Results and Comparison with the Competition

This subsection presents the results of the effectiveness of the ASR System, and compares them with the results obtained by other authors [4,5,6,7]. Unfortunately, very often, the implementation of such a comparison is an extremely difficult task, due to the different datasets of voices used by different authors. Moreover, even in the case of using the same dataset of voices, it is possible to use it differently during tests. However, below, the authors have appropriately adapted the dataset of voices that was used, to be able to compare the obtained results with the competition as faithfully as possible. For the tests, the TIMIT dataset was used, which was downsampled to 8 kS/s for the purposes of the tests. In addition, the size of the dataset used was appropriately limited, and the lengths of the training and test segments were adapted in accordance with the assumptions of the compared systems presented in Table 3. Some of the compared systems use the IR (Identification Rate) as a comparative parameter, and some use EER.

### 4.6. Results of Genetic Selection of Distinctive Features and Genetic Optimization of Internal Parameters of the ASR System

The genetic selection of distinctive features was one of the key steps in the development of the ASR System. Due to the fact that the results concerning this issue have already been partially published in [27,39], Table 4 presents only the profit obtained for the ASR System from the application of this method. The initial set of distinctive features were 30 MFCC features and 30 weighted cepstral features.

The genetic optimization of the internal parameters of the ASR System was one of the most time-consuming stages of the research. This research has not yet been published by the authors in any article. The 14 internal parameters of this ASR System listed in Table 5 were optimized.

The datasets used during the experiments included two commercial datasets (NIST 2002 SRE [3] and TIMIT [8]) and three proprietary datasets created for the purpose of the optimization and verification of the System described here (intonation dataset [21], dataset with recordings of digits and numbers [31], and multisession vocal dataset [39]). The total number of voices used for the following experiment is 1160.

Optimization took place in two stages. The first stage involved a rough optimization using the proprietary version of the divide-and-conquer algorithm, which assumed the calculation of values in all sub-divisions up to the third level of division, at each iteration, so as not to accidentally abandon an important area of solutions, sometimes rejected in the standard version of the method. Thanks to this part of the optimization, it was possible to determine the rational range of variability of the individual variables prepared for the second optimization stage. Moreover, thanks to the modified divide-and-conquer method, the correct identification in the group of 1160 speakers improved from 999 (86.12%) to 1099 (94.74%).

The second stage of optimization was global optimization using a genetic algorithm. Thanks to this approach, it was possible to consider all variables simultaneously, which allowed for optimal results to be obtained from a global perspective. For optimization purposes, an appropriately adapted function of the Optimalization Toolbox of the MATLAB environment was used. Each variable was limited by the range of variation established in the previous optimization stage.

**Figure 9:**
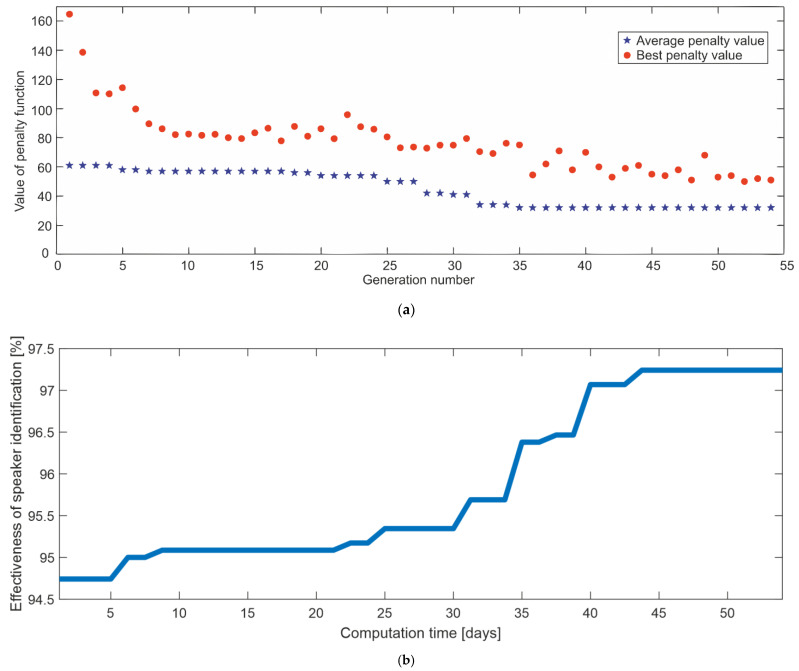
Results of the optimization of the internal parameters of the ASR System with the use of a genetic algorithm, (**a**)—value of the penalty function in relation to the number of generations; (**b**)—Effectiveness of speaker identification in relation to the calculation time.

Moreover, according to the assumption of the optimization function “ga” in the MATLAB environment, a value of 100 was assumed as the population size for the 14 variables. The genetic algorithm was stopped if the maximum number of iterations was reached (200 generations were assumed), or if there was no appropriate increase in the adaptation of the best individual for a given number of generations (20 generations were adopted). The optimization results are presented in Figure 9, (a)—value of the penalty function in relation to the number of generations of the genetic algorithm, (b)—effectiveness of speaker identification in relation to the calculation time of the genetic algorithm. A particularly important element was the use of the parameter values optimized in the first stage as the so-called “super individual” in the first generation of genetic optimization. This allowed for the commencement of evolutionary activities, taking into account a highly adapted individual, which significantly translated into the speed of obtaining further well-adapted descendants. This is of great importance, especially in the case of processing a large dataset, and the considered total dataset of voices is one of these.

The optimization shown in Figure 9 was stopped due to a lack of improvement in the fitness of the best individual for 20 generations.

It can be seen that the use of global optimization with the genetic algorithm allowed for a reduction in the number of misidentified speakers from 61 to 32 in the dataset of 1160 voices. This resulted in 1128 correct identifications, i.e., 97.24%.

The presented optimization results show that, despite the fine-tuning of individual parameters, only their global optimization allowed the highest possible results of the effectiveness of speaker recognition to be obtained. This is due to the fact that the parameters are strongly correlated with each other, and changing one of them essentially affects the others.

## 5. Conclusions

The authors have presented an automated speaker-recognition system that enables the effective identification and verification of speech signals featuring degraded quality similar to the ones that we deal with during telephone transmission. Some of the current literature dedicated to speaker recognition has enabled the comparison of the obtained results of speaker identification/verification due to the application of the same commercial datasets of voices. Moreover, the datasets should be tested in the same way so that a comparison of ASR Systems can be reliable, which often turns out to be difficult due to the different methods of testing by many authors of articles. For example, in the light of research conducted on the basis of the TIMIT [4,5,6,7] dataset, the results achieved by the authors are at least satisfactory, as they allow for the achievement of a higher IR level and a lowering of the EER.

The system was optimized over the course of a long-term optimization with the use of a genetic algorithm. Due to the abundance of voices in the datasets used, one can assume with large probability that the obtained parameters are universal and are not overly adapted to train data. Numerous tests carried out by the authors, including the use of speech codecs, give a clear message that the system is fully prepared for commercialization. In the final version of the system, the effectiveness of correct speaker identification in the open set amounted to 91.6 % with a dataset of 1529 voices.

At the stage of developing the ASR System concerned, the authors conducted its multi-criteria optimization (including the use of a genetic algorithm), to adapt it to work effectively under conditions that take account of the limitations imposed by a telephone channel, which is its undoubted asset. A unique vector of distinctive features is a key part of the system. The vector results from appropriate feature generation, selection, and normalization. One should also mention the application of a productive classification with the use of GMMs, which has recently been of prime importance in voice biometry systems.

The use of a genetic algorithm for the selection of features, along with the proprietary fitting function, has allowed not only a significant increase in the effectiveness of the ASR system, but also a reduction in data. Currently, a single speaker’s voice model is only 700 bytes. These elements, according to the authors, constitute the main aspect of the innovation of this ASR System.

The ASR System concerned certainly needs continuous improvement, so as to meet the current challenges faced by voice biometry, such as, e.g., a continuous increase in the system’s resistance to impersonation attempts, including with the use of deepfake technology, which is becoming more and more hazardous for cyberspace. However, one can conclude that the goal set by the authors, namely, to achieve a level of voice signal processing such that one can create memory-economical voice models that enable highly effective speaker identification/verification, has been achieved.

## Figures and Tables

**Table 1:** Results of speaker equal error rate in particular voice datasets.

Name of the Voice Dataset	NIST	TIMIT	Proprietary (Digits and Numbers)	Proprietary (Intonation Differentiation)	Proprietary (Multisession)	SNUV	Proprietary (i.e., Throat Microphone)	VCTK	Total Dataset
Dataset size (number of voices)	330	630	100	50	50	210	50	109	1529
EER (%)	1.52	0.48	1.00	0.00	6.00	3.33	4.00	0.92	2.03

**Table 2:** Results of speaker identification (I) and verification (V) effectiveness for the common coding standards.

Training\Testing	Uncoded		G.711		GSM 06.10		G.723.1		SPEEX	
	I	V	I	V	I	V	I	V	I	V
Uncoded	**96.3**	**98.0**	95.7	97.7	82.3	96.1	80.8	95.8	90.5	96.6
G.711	96.0	98.1	**96.1**	**97.9**	84.0	96.3	79.9	96.0	91.8	96.9
GSM 06.10	85.9	96.5	86.9	96.7	**93.5**	**97.3**	72.5	93.9	84.4	95.5
G.723.1	84.5	96.4	84.2	96.0	71.6	93.9	**92.5**	**97.2**	86.3	95.9
SPEEX	92.6	97.7	93.1	97.4	83.1	96.2	85.2	96.6	**95.2**	**97.6**

**Table 3:** Comparison of the results, effectiveness of ASR Systems using the TIMIT dataset.

Dataset Size	Training Signal	Test Signal	Comparative Parameter	Result for This ASR System	The Result for the Compared ASR System	Methods Used	References of the ASR System Compared
100 random voices from the “test” folder	5 utterances	5 utterances	IR	99.00%	80.00%	MFCC, GMM-UBM	[4]
38 random voices (19 male, 19 female) from the “test” folder	No data—7 utterances were assumed	no data—3 utterances were assumed	IR	100.00%	82.84%	MFCC, LFA-SVM	[5]
130 random voices from the “train” folder	8 utterances	2 utterances	EER	Uncoded speech: 0.77% Synthesized speech (G.729): 2.31%	Uncoded speech: 0.91% Synthesized speech (G.729): 12.50%	MFCC, LDA, GPLDA	[6]
124 random voices from the “test” folder	6 utterances, limited to 7 s	4 utterances, limited to 7 s	IR	Clean speech: 98.39% AWGN noise (30 dB): 95.16%	Clean speech: 97.52% AWGN noise (30 dB): 86.70%	Multi-taper (MFCC + PNCC), ELM	[7]

**Table 4:** Influence of genetic feature selection on the ASR System.

Parameter	Value
Number of reduced individual features	38
Increase of IR for NIST SRE 2002 dataset	21%
Increase of IR TIMIT dataset	20%

**Table 5:** A set of internal ASR System parameters subject to genetic optimization.

Stage	Parameter
Silence cutting	Frame length
	Frame overlap
	Threshold
Signal filtration	High-pass filter row
	Cut-off frequency
Cepstrum filtration	Low-pass filter row
	Cut-off frequency
Fundamental frequency filtration	Minimum fundamental frequency
	Maximum fundamental frequency
Feature generation	Frame length
	Frame overlap
	Voicing threshold
	Power threshold
	Fundamental frequency difference threshold

## Data Availability

Not applicable.
